# Clinical Outcomes of Open-Wedge Varus Derotation Osteotomy in Legg-Calve-Perthes Disease Among 6-12-Year-Old Children

**DOI:** 10.7759/cureus.41144

**Published:** 2023-06-29

**Authors:** Appuchip Gumin Tayeng, Aniruddha Sengupta, Priyajit Chaterjee

**Affiliations:** 1 Department of Orthopaedics, All India Institute of Medical Sciences, Guwahati, Guwahati, IND; 2 Department of Orthopaedics, Medical College and Hospital, Kolkata, IND; 3 Department of Orthopaedics, Diamond Harbour Goverment Medical College and Hospital, Diamond Harbour, IND

**Keywords:** legg-calve-perthes disease, catterall classification, harris hip score, varus derotation open wedge osteotomy, late presentation, children

## Abstract

Background

Legg-Calve-Perthes (LCP) disease during early childhood might lead to impaired blood circulation to the femoral head causing osteoarthritis and avascular necrosis of the femoral head. In most cases of children diagnosed after the age of nine years, the progression of LCP disease is associated with poor outcomes causing disability. This study aimed to evaluate the clinical and radiological outcomes of varus derotation osteotomy in children with LCD disease with a late presentation at 6-12 years of age.

Methodology

This prospective study was performed among 25 6-12-year-old children who were diagnosed with LCD disease. Children were classified per Herring’s Lateral Pillar and Catterall classification. They were treated with open-wedge varus derotation osteotomy, and clinical, radiological, and functional parameters were assessed postoperatively. The postoperative grading was done using the Catterall classification.

Results

In this study, significant improvement in the postoperative mean Harris Hip Score (82.96 ± 4.3 vs. 85.36 ± 3.75; p = 0.000) was observed. In addition, the mean muscle mass reduced postoperatively compared to the preoperative mass (2.64 ± 3.82 vs. 8.52 ± 3.73 mm; p = 0.000). Based on the Catterall classification, 56% of children showed good outcomes, and 36% showed fair outcomes. Regarding complications, 20% had postoperative infections.

Conclusions

In our study, open-wedge varus derotation osteotomy showed improved clinical and radiological outcomes in children with late presentation of LCD disease.

## Introduction

Legg-Calve-Perthes (LCP) disease is a rare painful disorder in children characterized by avascular necrosis of the femoral head [1]. However if not properly managed at an early stage, it leads to femoral head deformation, followed by early osteoarthritis, flattening, and subluxation of the hip joint later in life. The age, grade of LCP, sphericity of the femoral head, a pattern of effect per Herring classification, and the presence of lateral pillar type C are the important factors that affect the prognosis [2]. The important objective of the therapy is to prevent femoral head distortion before the revascularization stage [3]. Femoral head containment within the acetabular cavity reduces the mechanical load and permits the normal growth of the head and acetabular cavity and maintains joint congruency. This can be achieved by conservative approaches such as traction, minimizing the weight-bearing, and braces, or by surgical approaches. Based on earlier studies, surgical containment by varus derotation osteotomy (VDRO) and Salter innominate osteotomy display comparable outcomes, but the latter has the potential for a neurovascular injury [4]. The advantage of VDRO is that it elicits varus angulation and corrects the rotation which aids the femoral head coverage toward the acetabulum and produces medial and anterior redirection, thus preventing the lateral dislocation of the femoral head, and in addition, the joint congruity end reduces femoroacetabular impingements [2].

The progression of LCP is not severe in children aged less than six years and can be managed through conservative treatment [5,6]. However, in children aged six to nine years, correction of the femoral heads needs a surgical approach [7-11]. However, in children aged more than nine years, surgical management is challenging and is associated with poor prognosis [11]. Earlier reports have shown promising results wherein surgical containment by VRDO showed improved outcomes in children aged nine to ten years compared to conservative treatment [11]. However, in some studies reporting conflicting results, surgical containment in LCP disease in children aged more than eight years showed poor outcomes [12,13].

Hence, this study aimed to assess the clinical and radiological outcomes of VDRO with respect to the Herring classification for interobserver reliability and the Catterall classification for prognosis in children with LCP disease. In addition, limb length discrepancy, restriction of range of motion, atrophy of thigh muscles of the affected side, pain reduction, and improvement in the quality of life were also evaluated.

## Materials and methods

This prospective study was conducted among 25 children aged 6-12 years with LCD disease. Children who presented to outpatient clinics or were admitted directly for operative management at the pediatrics orthopedic ward of Medical College and Hospital, Kolkata from January 1, 2018, to May 31, 2019 (17 months duration) were included in this study. All children were treated with VDRO. The study was conducted after obtaining approval from the institutional ethical committee of Medical College and Hospital, Kolkata (approval number: MC/KOL/IEC/NON-SPON/1034/11/2017).

Inclusion criteria

Children aged 6-12 years of both sexes who were diagnosed with LCP disease, Herring lateral pillar types B and C and/or Catterall types II, III, and IV, and whose parents/guardians of the children were willing to provide consent for the study were included. Children with LCP disease, Herring lateral pillar type A and/or Catterall type I, any associated disease along with LCP disease, Herring lateral pillar types B and C and/or Catterall types II, III, IV, aged less than six years and more than 12 years, those who were more inclined to conservative treatment, and those with other comorbidities were excluded from the study.

Study design

This prospective study emphasized patients’ functional and radiological status. Operative intervention was performed in due course and children were followed up postoperatively for nine months. A detailed history was obtained from patients regarding their symptoms, and the preoperative Harris Hip Score was calculated for each patient. Anteroposterior as well as frog-leg lateral views of the hip were obtained. The children were categorized per Herring’s lateral pillar and Catterall classification.

Surgical procedures

After a proper preoperative assessment, all children were managed with VDRO. Children were prepared 24 hours before the surgery. Preoperative procedures such as cleaning the operating limb and prior antibiotic administration were done according to the standard protocol. All cases were operated under general anesthesia and/or spinal anesthesia. The child was positioned supine on the radiolucent operating table. After antiseptic application and draping, the bony landmarks were marked. The open-wedge VDRO of the proximal femur was done through a lateral approach. The skin was incised from the base of the greater trochanter to about 10-15 cm distally. The subcutaneous tissue and tensor fascia lata were incised in the same line. The vastus lateralis muscle was split along its fibers and retracted to reach the proximal femur. With the help of an oscillating saw under fluoroscopic guidance, the osteotomy was made aiming for 15-20 degrees of varus correction and a neck shaft angle of 110- 120 degrees intraoperatively with regular irrigation with normal saline to prevent heat necrosis. External rotation correction of 15 to 20 degrees was performed at the osteotomy site, and the osteotomy site was fixed with a 3.5 mm molded small dynamic compression plate along with screws. Subsequently, the wound was thoroughly irrigated with normal saline, hemostasis was secured, a suction drain of appropriate size was applied, and the wound was covered in several sheets. The dressing was changed after 48 hours with drain removal. Stitches were removed 12 to 14 days postoperatively. Quadriceps and hamstring exercise training were performed 10-15 hours postoperatively after the pain had subsided. In non-cooperative children, a hip spica was done.

Postoperative follow-up

The first follow-up was done after two weeks and the second follow-up was done at six weeks. The patients were evaluated for pain, stiffness, range of motion, and Harris Hip Score, and the functional scores were determined. In addition, the postoperative neck-shaft angle (degrees) and limb length shortening were evaluated.

Functional results

Catterall’s postoperative classification was used to categorize the hips as good, fair, and poor depending on the range of hip motion, hip symptoms, and radiography [13,14].

Statistical analysis

The data were presented as mean ± SD. Preoperative and postoperative results were compared using the unpaired Student’s t-test. The association between categorical variables was determined using Fischer’s exact test. P-values <0.05 were considered statistically significant.

## Results

The mean age of the children was 8.20 ± 2.12 years (range = 6-12 years). Overall, 64% of cases were aged 6-8 years, and 36% of children were aged 9-12 years. Furthermore, 72% of the children were males, and 28% were females. Regarding involvement, 36% of children had right-sided involvement and 64% had left-sided involvement.

There was a significant improvement in the mean Harris Hip Score postoperatively when compared to the preoperative score (85.36 ± 3.75 vs. 82.96 ± 4.3; p = 0.000). There was a significant reduction in muscle wasting postoperatively compared to preoperatively (2.64 ± 3.82 vs. 8.52 ± 3.73 mm; p = 0.000) (Table 1).

**Table 1:** Harris Hip Score and muscle wasting after the surgery. *Pvalue<0.05, statistically significant

Variables (mean ± SD)	Preoperative	Postoperative	P-value
Harris Hip Score	82.96 ± 4.3	85.36 ± 3.75	0.000*
Muscle wasting (mm)	8.52 ± 3.73	2.64 ± 3.82	

Regarding outcomes, 56.0% of the children showed good outcomes, 36.0% of the children showed fair outcomes, and 8.0% of the children showed poor outcomes.

The mean age of the children with good outcomes was 7.86 ± 2.03 years, with fair outcomes was 8.11 ± 2.09 years, and with poor outcomes was 11 ± 1.41, which was not significant (p = 0.145). Regarding the association between gender and outcomes, 61.1% of male children showed good outcomes, 33.3% showed fair outcomes, and 5.6% showed poor outcomes. Among the female children, 42.9% showed good outcomes, 42.9% showed fair outcomes, and 14.3% showed poor outcomes, and this association was not significant (p = 0.525).

Of the children with lateral pillar B, 70.6% had good outcomes, 29.4% had fair outcomes, and none of the patients had poor outcomes. Of the children with lateral pillar C, 25% had good outcomes, 50% had fair outcomes, and 25% had poor outcomes. The association between the lateral pillar and outcomes was found to be significant (p = 0.034) (Table 2).

**Table 2:** Association of the lateral pillar with outcomes. *: P-value <0.05 is statistically significant

Lateral pillar	Good, N (%)	Fair, N (%)	Poor, N (%)	Total, N (%)	P-value
B	12 (70.6)	5 (29.4)	0 (0)	17 (100)	0.034*
C	2 (25)	4 (50)	2 (25)	8 (100)	
Total	14 (56)	9 (36)	2 (8)	25 (100)	

Among the patients in Catterall type II, 75% had good outcomes, 16.7% had fair outcomes, and none of the patients had poor outcomes. In patients with Catterall type III, 33.3% had good outcomes, 42.9% had fair outcomes, and 7.1% had poor outcomes. Meanwhile, in patients with Catterall type IV, 40% had good outcomes, 40% had fair outcomes, and 20% had poor outcomes. The association between Catterall type and outcome was found to be non-significant (p = 0.504) (Table 3).

**Table 3:** Association of Catterall type with outcomes. NS: statistically non-significant

Catterall type	Outcome			Total, N(%)	P-value
	Good, N (%)	Fair, N (%)	Poor, N (%)		
II	5 (75)	1 (16.7)	0 (0)	6 (100)	0.504^NS^
III	7 (33.3)	6 (42.9)	1 (7.1)	14 (100)	
IV	2 (40)	2 (40)	1 (20)	5 (100)	
Total	14 (68)	9 (36)	25 (8)	25 (100)	

Among the children in Stulberg class I, 91.7% had good outcomes, 8.3% had fair outcomes, and none of the children had poor outcomes. Of the children with Stulberg class II, 37.5% had good outcomes, 62.5% had fair outcomes, and none of the children had poor outcomes. Of the children with Stulberg class III, all three (100%) children had fair outcomes. Of the children with Stulberg class IV, all two (100%) children had poor outcomes, and it was found to be significant (p = 0.000) (Table 4).

**Table 4:** Association of Stulberg class with outcomes. *: P-value <0.05, statistically significant

Stulberg class	N (%)	N (%)	N (%)	Total, N (%)	P-value
I	11 (91.7)	1 (8.3)	0 (0)	12 (100)	0.000*
II	3 (37.5)	5 (62.5)	0 (0)	8 (100)	
III	0 (0)	3 (100)	0 (0)	3 (100)	
IV	0 (0)	0 (0)	2 (100)	2 (100)	
Total	14 (56)	9 (36)	2 (8)	25 (100)	

The association of Stulberg class with the lateral pillar is shown in Table 5. Among the children in Stulberg class I, 83.3% were in lateral pillar B, and 16.7% were in lateral pillar B. Among the children with Stulberg class II, 75% were in lateral pillar B, and 25.5% were in lateral pillar C. Of the children with Stulberg class III, 33.3% were in lateral pillar B, and 66.7% were in Lateral pillar C. Of the children with Stulberg class IV, all two (100%) children were in lateral pillar C. In this study, the association between Stulberg class with lateral pillar type was found to be significant (p = 0.000).

**Table 5:** Association of Stulberg class with lateral pillar. *: P-value <0.05, statistically significant

Stulberg class	Lateral pillar B, N (%)	Lateral pillar C, N (%)	Total, N (%)	P-value
I	10 (83.3)	2 (16.7)	12 (100)	0.000*
II	6 (75)	2 (25.5)	8 (100)	
III	1 (33.3)	2 (66.7)	3 (100)	
IV	0 (0)	2 (100)	2 (100)	
Total	17 (68)	8 (320)	25 (100)	

The association of Stulberg class with Catterall type is shown in Table 6. Among the children in Stulberg class I, 25% were in Catterall type II, 58.3% were in Catterall type III, and 16.5% were in Catterall type IV. In Stulberg Class II, 37.5% were in Catterall types II and III, and 25% were in Catterall type IV. In Stulberg class III, all three (100%) children were in Catterall type III, and in Stulberg Class IV, 50% of the children were in Catterall types III and IV. There was a significant association between Stulberg class and Catterall type (p = 0.000).

**Table 6:** Association of Stulberg class with Catterall type. *: P-value <0.05, statistically significant

Stulberg class	Catterall type				P-value
	II, N (%)	III, N (%)	IV, N (%)	Total, N (%)	
I	3 (25)	7 (58.3)	2 (16.5)	12 (100)	0.000*
II	3 (37.5)	3 (37.5)	2 (25)	8 (100)	
III	0 (0)	3 (100)	0 (0)	3 (100)	
IV	0 (0)	1 (50)	1 (50)	2 (100)	
Total	6 (24)	14 (56)	5 (20)	25 (100)	

The mean limb length shortening in children during the postoperative period was 7.96 ± 5.33 mm (range = 0-15 mm). The mean postoperative neck-shaft angle was 125.84 ± 2.23 degrees (range = 121-129 degrees) (Table 7).

**Table 7:** Postoperative neck-shaft angle and limb length shortening among the children.

Variables	Mean ± SD	Minimum	Maximum
Limb length shortening (mm)	7.96 ± 5.33	0	15
Neck-shaft angle (degrees)	125.84 ± 2.23	121	129

In this study, limping was present in 20% of the children, and 20% of the children had postoperative infections.

## Discussion

Open-wedge VDRO of the femur is a well-documented surgical technique for the management of LCP disease. Although the precise etiology is unknown, the age at presentation and the degree of participation of the femoral head are the greatest independent prognostic factors. A prospective study on the consequence of VDRO in LCP disease was performed among 25 patients in this study.

In this study, 68% (17 cases) of patients belonged to Herring’s lateral pillar group B and 32% (eight cases) of children belonged to group C, which is similar to the study done by Kumar et al. where 67% (59 cases) of children belonged to group B and 33% (29 cases) of children belonged to group C [15]. Regardless of classification, therapy aims to re-establish the sphericity, epiphyseal height, and congruity of the joint in the long term, which is the chief prognostic factor. Research to quantify the morphology of the femoral head and the acetabulum continues to progress. Numerous studies have followed the Moses index in their research [16]. Nevertheless, it is not probable to measure some femoral heads, which are not circular enough to fit the outline of the Moses ring, using the Moses index, as reported by Dickens et al. In their research, they found it difficult to fit Moses rings to every femoral head. Therefore, we avoided Moses rings [17].

In this study, 56% of patients had good outcomes, 36% of patients had fair outcomes, and 8% of patients had poor outcomes, which is in line with the study reported by Kumar et al. In the study by Kumar et al., 116 (51%) patients had good hips, 40% had fair hips, and 9% had poor hips, and the patients with poor outcomes (Stulberg V) were patients with Herring class C involvement and more than nine years of age [16]. In another study conducted among 88 patients, 75% had good, 21.6% had fair, and 3.4% had poor outcomes [15]. In their study, Herring et al. reported that the lateral pillar arrangement and age at the time of disease onset showed a good correlation with LCP disease outcome. Children aged more than eight years at disease onset with hip in the lateral pillar B or B/C classification showed better outcomes with surgical procedures compared to non-operative management. Children with lateral pillar B hips and aged below eight years at disease onset showed effective outcomes irrespective of the treatment, either surgical or non-surgical, and children with lateral pillar C hips, irrespective of age, showed poor outcomes whether treated surgically or non-surgically [7].

In this study, the mean age at onset of disease was 8.20 years, and the majority of the children (64%) were aged 6-8 years, and 36% were aged 9-12 years. Previous studies showed the age at onset of 8.14 years [18] and 9.2 years [2]. They also reported that children aged less than 10 years showed better outcomes compared to patients aged more than 10 years. In another study done by Noonan et al., among the patients who had undergone femoral varus osteotomy, the children aged 9-10 years showed better outcomes compared to children aged more than 10 years [11]. In our study, a male preponderance was observed, and the involvement of LCP disease was more on the left side compared to the right side. In addition, the Harris Hip Score increased and muscle wasting decreased postoperatively compared to preoperatively which was significant.

In this study, there was no significant association between age and outcome, and the outcome was poor in children with a mean age of 11 years. Further, there was no significant association between gender and outcome, but the incidence of good outcomes was higher in males compared to females (61.1% vs 42.9%); however, it was not significant. In our study, the majority of the children belonged to Herring’s lateral pillar B (68%) and C (32%), which is consistent with the study reported by Kumar et al. where 67% (59) of children belonged to lateral pillar B, and 33% (29) of children belonged to lateral pillar C [15]. In this study, based on the lateral pillar category, 56% of patients showed good outcomes, 36% had fair outcomes, and 8% showed poor outcomes, which is in line with the study by Kumar et al. where 75% had good outcomes, 21.6% had fair outcomes, and 3.4% had poor outcomes [15].

In our study, 70.6% of the children in group B had good outcomes, and 29.4% of the children had fair outcomes. None of the children in group B had poor outcomes. Moreover, 25% of the children in group C had good outcomes, 50.0% of the children had fair outcomes, and 25% of the children had poor outcomes. Herring et al. found that the lateral pillar classification and age at the time of onset of the illness are intensely associated with the outcome in children with LCP disease. Children aged eight years at the time of disease onset showed an improved outcome with surgical therapy than with non-operative therapy with favorable outcomes unrelated to therapy [19]. In the study done by Saini et al., 17 of the 23 children (73.9%) in the Herring lateral pillar group B showed good outcomes, 26.08% had fair outcomes, and none of the patients had poor outcomes. In group C, 27% had good outcomes, 63.63% had fair outcomes, and 9.09% had poor outcomes [2]. Similar results were reported by Kumar et al. with a correlation between the lateral pillar group and outcomes [15].

Saini et al. have recommended VDRO to be an effective surgical procedure for LCP disease, specifically among children aged less than 10 years. The study included 45 children with a mean age of 9.2 years, belonging to Herring’s groups B and C, with good results in 23, fair results in 20, and poor results in two children [2]. Noonan et al. also reported comparable findings. VDRO was satisfactory in children aged less than 10 years [11].

Clinical outcomes in our cases were recorded by measuring the Harris Hip Score. In this study, none of the children had fixed deformity, and all children could sit cross-legged and could squat postoperatively because the mean postoperative Harris Hip Score was 85.36, indicating good hip function. Coates et al. reported a mean Harris Hip Score of 97.8, and Joshi et al. reported a mean Harris Hip Score of 93.2 [20,21], which is consistent with our study findings.

In this study, Stulberg class I had 48% of cases, class II had 32% of cases, class III had 12%, and class IV had 8% of cases. Similar to our report, Beer et al. reported Stulberg class I hips in 19.5%, Stulberg class II hips in 36.6%, Stulberg class III hips in 19.5%, Stulberg class IV hips in 22%, and Stulberg class V hips in 2.4% of the children [22]. Likewise in the study by Herring et al., 51% of hips were Stulberg classes I and II, 34% were class III, and 15% were classes IV and V [19].

Wiig et al. reported an association between Catterall classification and lateral pillar groups. They documented a significant relationship (p < 0.001) between Catterall classification and radiographic outcomes during the five-year follow-up. Among 44 hips, 84% showed femoral head participation, less than 50% in Catterall groups I and II resulted in Stulberg groups I to II, while none of the cases were in groups IV or V. The results showed worse outcomes, with more than 50% of hips having femoral head necrosis, that is, Catterall groups III and IV, where 60 of 314 (19%) were in Stulberg IV or V [23].

Among the children in Stulberg class I, 83.3% were in lateral pillar B, and 16.7% were in lateral pillar C. In children with Stulberg class II, 75% were in lateral pillar B, and 25.5% were in lateral pillar C. In children with Stulberg class III, 33.3% were in lateral pillar B, and 66.7% were in lateral pillar C. In children with Stulberg class IV, all two (100%) children were in lateral pillar C. In this study, the association between Stulberg class with lateral pillar type was found to be significant (p = 0.000).

Among the children in Stulberg class I, 25% were in Catterall type II, 58.3% were in Catterall type III, and 16.5% were in Catterall type IV. In Stulberg class II, 37.5% were in Catterall types II and III, and 25% were in Catterall type IV. In Stulberg class III, all three (100%) children were in Catterall type III, and in Stulberg class IV, 50% of the children were in Catterall types III and IV. There was a significant association between Stulberg class and Catterall type (p = 0.000).

The mean limb length shortening of the participants was 7.96 mm (range = 0-15 mm), and five patients experienced limping due to the resultant short limb gait. Saini et al. reported limb length shortening in 12 patients with a mean shortening of 11 mm (range = 6-15 mm), and 5 patients had associated short limb gait [2]. All femoral heads were contained within the femoral head, and the mean postoperative neck-shaft angle in our study was 125.84 degrees (range = 121-129 degrees), which is comparable to the results documented by Saini et al. with a mean neck-shaft angle of 117 degrees (range = 102-125 degrees) [2].

In this study, there was a significant decrease in muscle wasting postoperatively from 8.52 mm to 2.64 mm, which reflects that the improvement in hip function disuse atrophy of the muscles decreased. Management of children aged more than eight years remains provocative, and only a few studies have established that VDRO is acceptable in children aged less than ten years in contrast to natural history or non-containment techniques [24-26]. Bayliss et al. documented that in such age groups, earlier containment of the head enhanced the prognosis [27]. Five patients in our study developed postoperative infections which were treated with intravenous antibiotics, following which the infections resolved without any further complications.

The limitations of our study were its small sample size, illiteracy in some cases, and short follow-up duration. A lengthier follow-up period is necessary to assess whether the beneficial effects of VDRO are sustained over a period of time. The absence of a control group is another major limitation of this study.

## Conclusions

VDRO is an efficient mode of treatment for children with Herring’s lateral pillar group B and Catterall groups II and III. It is also effective in some cases with Herring’s lateral pillar group C and Catterall group IV. The degree of interobserver reliability of the Stulberg scale with the Caterall scale is higher, and the validity and reliability of the lateral pilar scale are higher when compared to the Caterall scale. These outcomes are also related to the age of the patient with favorable outcomes for children aged less than 8 years. Limb shortening is a known complication and a common reflection that many children endure improved shortening than lengthening which is usually less than 20 mm.
